# Global analysis of soybean bZIP gene family under stress and identification of salt-stress-responsive candidate genes

**DOI:** 10.3389/fpls.2026.1806221

**Published:** 2026-03-20

**Authors:** Enqiang Zhou, Mengnan Yao, Yongqiang Wang, Dong Xue, Yao Zhou, Xuejun Wang, Yuxin Shi, Yuxiang Zhu, Zongdi Li, Na Zhao, Kaihua Wang, Bo Li, Chunyan Gu, Yamei Miao, Libin Wei

**Affiliations:** Department of Economic Crops, Jiangsu Yanjiang Institute of Agricultural Science, Nantong, China

**Keywords:** abiotic stress, GmbZIP, salt stress, shade stress, soybean, waterlogging

## Abstract

Basic leucine zipper (bZIP) genes are extensively involved in various physiological processes, including seed development, light signal regulation, and stress responses. However, the regulatory roles of soybean *GmbZIP* genes in mediating abiotic stress responses remain poorly understood. To systematically identify abiotic stress-responsive *GmbZIP* genes in soybean and elucidate their evolutionary relationships, this study conducted a genome-wide identification and evolutionary analysis of the GmbZIP gene family using bioinformatics methods, combined with transcriptome data from waterlogging, salt, and shade stress treatments. Furthermore, comparative genomics was employed to screen for *GmbZIP* genes potentially associated with salt stress response, followed by protein-protein interaction network analysis and experimental validation via qRT-PCR. The results showed that a total of 92 *GmbZIP* genes were identified from the stress-related transcriptome datasets. Phylogenetic analysis classified them into 13 distinct subfamilies, each exhibiting significant evolutionary divergence and diversity in protein physicochemical properties, gene structures, and conserved motifs. Collinearity analysis revealed 143, 122, 104, 49, and 30 collinear gene pairs between soybean and *Medicago truncatula* (*M. truncatula*), *Solanum lycopersicum* (*S. lycopersicum*), *Arabidopsis thaliana*, *Oryza sativa* (*O. sativa*), and *Zea mays* (*Z. mays*), respectively. Segmental duplication was identified as the primary driver of the expansion of this gene family, with purifying selection playing a dominant role during its evolution. KEGG enrichment analysis indicated that *GmbZIP* genes are predominantly enriched in plant hormone signal transduction pathways. Consistent with this finding, cis-regulatory element analysis of promoters showed that 98.9% of the *GmbZIP* genes contain five types of hormone-responsive elements. Through comparative genomic screening, 12 candidate *GmbZIP* genes potentially involved in salt stress response were identified. Both protein-protein interaction network prediction and qRT-PCR expression validation support that these genes may be involved in the regulation of salt stress. In summary, this study presents the first systematic identification and evolutionary analysis of the abiotic stress-responsive GmbZIP gene family in soybean, providing an important foundation for further investigation into the functional mechanisms of these genes in plant abiotic stress responses.

## 1 Introduction

Plants frequently encounter various abiotic stresses such as extreme temperatures, shade, waterlogging, drought, and salinity during growth and development. These stresses significantly impair physiological processes and morphogenesis, ultimately leading to reduced productivity (Rahman et al., 2025). To cope with these adversities, plants have evolved multiple adaptive strategies, which are often achieved through sophisticated signal transduction pathways. Among them, transcription factors (TFs) play a pivotal role in the signal networks, and their core function is to transmit perceived stress signals to downstream stress response genes, thereby modulating plant tolerance to abiotic stresses (He et al., 2020). Common stress-related TF families include MYB, bHLH, WRKY, and bZIP. Notably, the bZIP TF family represents one of the largest and most diverse TF families in plants (Liu et al., 2023).

All members of the bZIP TF family contain a highly conserved bZIP domain (Dröge-Laser et al., 2018). This domain is composed of 60–80 amino acids, consists of two functionally distinct regions: the C-terminal is a basic region rich in arginine and lysine, which is composed of about 20 amino acid residues, and its conserved nuclear localization sequence N-x7-R/K can specifically recognize and bind DNA cis-acting elements (Nijhawan et al., 2008); The N-terminal is leucine zipper domain, and its characteristic sequence L-x6-L-x6-L is formed by periodic repetition of leucine or other hydrophobic amino acids, which mediates homologous or heterodimerization of bZIP protein (Zhang et al., 2023). To date, genome-wide identification and analysis of bZIP TFs have been completed in various plant species, including pecan (Jiang et al., 2023), wheat (Liang et al., 2022), faba bean (Huang et al., 2023), soybean (Wang et al., 2015; Zhang et al., 2018), and pea (Wu et al., 2023b). These studies have laid a theoretical foundation for elucidating the evolution and function of bZIP genes.

Research indicates that bZIP TFs are widely involved in regulating plant growth and development, such as root architecture establishment (Ma et al., 2018), starch biosynthesis (Song et al., 2020), and seed development (Yang et al., 2023b). Concurrently, this family plays a crucial role in plant responses to abiotic stresses including salt stress, shade, waterlogging, drought, and low temperature. For instance, overexpression of *TabZIP15* enhances salt tolerance in wheat (Bi et al., 2021). *SlbZIP1* in tomato improves salt tolerance by activating genes associated with ABA biosynthesis and signal transduction (Zhu et al., 2018). Overexpression of *AtAREB1* in soybean simultaneously enhances plant adaptation to drought and waterlogging (Fuhrmann-Aoyagi et al., 2021), while *GmbZIP71–4* negatively regulates waterlogging stress responses via the ABA signaling pathway (Lin et al., 2024). *MdbZIP80* in apple enhances drought resistance by negatively regulating cytokinin signaling (Feng et al., 2021). *Arabidopsis bZIP59* has been shown to negatively regulate shade-induced hypocotyl elongation (Li et al., 2025). In rice, *OsNAC5* cooperates with *OsABI5* to positively regulate low-temperature tolerance during germination and seedling stages (Li et al., 2024b).

Soybean (*Glycine max*) serves as a vital global source of protein and oil, playing a significant role in ensuring food security and nutritional health. However, soybean production is often constrained by various environmental stresses, including salinity, high temperature, waterlogging, and drought, which markedly affect its growth, development, and final yield. Moreover, soybean is a light-loving crop. In order to improve land use efficiency and economic benefits, soybean is frequently intercropped with maize, sorghum, sunflower or fruit trees, thereby resulting in shade stress. Currently, no study has conducted a genome-wide identification and evolutionary analysis of the bZIP TF family in soybean expressed under abiotic stresses. Therefore, this study aims to perform genome-wide identification of soybean *GmbZIP* genes, screen differentially expressed members responsive to abiotic stresses, and conduct further analyses including phylogenetic evolution, Ka/Ks analysis, prediction of promoter cis-elements, expression validation of candidate genes, and protein-protein interaction network construction. These efforts seek to elucidate the potential roles of soybean *GmbZIP* genes in abiotic stress responses and provide a theoretical basis for the genetic improvement of stress tolerance in soybean.

## 2 Materials and methods

### 2.1 Experimental materials and stress treatments

The waterlogging-tolerant soybean variety Chuanong 5 (CN-5) and waterlogging-sensitive variety Suxia 19-787 (SX19-787) were selected as experimental materials for the waterlogging stress study. The treatment group consisted of seeds subjected to 36 hours of waterlogging stress (T), while the control group used untreated seeds (CK). Subsequently, a 24-hour rolled paper germination test was conducted, and normally germinated seeds were selected for transcriptome sequencing (Wang et al., 2025). In the salt stress experiment, the salt-tolerant soybean variety Xudou 18 (XD18) and the salt-sensitive soybean variety Zhexia 2022 (ZX2022) were used as experimental materials. Both materials were cultivated in pure vermiculite in a light incubator until the seedlings grew to the stage with two fully expanded true leaves. Subsequently, the plants were removed for salt stress treatment: the roots of plants in the treatment group were soaked in a 6‰ NaCl solution, while those in the control group were soaked in normal water solution. Leaf tissues were collected for transcriptome sequencing after 14 days of treatment. In the shading stress experiment, shading-tolerant soybean material 275 (275) and shading-sensitive soybean material 235 (235) were used as experimental materials. In the field environment, shading nets were used to reduce light intensity by 30% to simulate shading stress. Plants in the treatment group were continuously shaded from the early seedling stage to the full flowering stage, while plants in the control group were grown under natural light conditions. At the full flowering stage, shoot apical meristems were collected separately from the treatment and control groups for transcriptome sequencing analysis. Three biological replicates were performed for sampling in all three experiments mentioned above.

### 2.2 Identification of soybean GmbZIP family members and stress differential *GmbZIP*

In this study, soybean genomic data (Wm82.a2.v1) was downloaded from the SoyBase genome database (https://legacy.soybase.org/) to identify members of the GmbZIP gene family. First, the bZIP gene family profile (PF00170) was obtained from the Pfam database (http://pfam.xfam.org/), and its corresponding hidden Markov model (HMM) file was downloaded (Finn et al., 2014). Using TBtools software (Chen et al., 2020), we performed an initial screening of the soybean genome to identify potential *GmbZIP* genes containing the conserved bZIP domain. The candidate *GmbZIP* sequences were then submitted to the NCBI Conserved Domain Database (CDD, https://www.ncbi.nlm.nih.gov/cdd) for verification of the bZIP conserved domain. Redundant, duplicated, and incompletely annotated sequences were removed, and the remaining genes were considered valid members of the soybean GmbZIP gene family. To identify stress-responsive *GmbZIP* genes, we analyzed their expression patterns across different stress treatments using DESeq2 (Love et al., 2014). Differentially expressed genes (DEGs) were screened based on fold change (FC) and false discovery rate (FDR), with the thresholds set at |log2(FC)| ≥ 1 and FDR < 0.05 (Sajjad et al., 2024).

### 2.3 Analysis of *GmbZIP* gene sequence characteristics

The amino acid number, molecular weight, theoretical isoelectric point (pI), instability index, aliphatic index, and grand average of hydropathicity (GRAVY) of GmbZIP family members were calculated using TBtools software. Subcellular localization of GmbZIP proteins was predicted using the online tool Cell-PLoc 2.0 (http://www.csbio.sjtu.edu.cn/bioinf/Cell-PLoc-2/). Protein sequences of *GmbZIP* genes were submitted to MEGA 7.0 software for multiple sequence alignment (Kumar et al., 2016), followed by phylogenetic tree construction using the maximum likelihood (ML) method with bootstrap values calculated from 1000 replicates. The conserved domains of *GmbZIP* genes were identified through the NCBI CDD. Protein sequences were submitted to the MEME suite (https://meme-suite.org/meme/tools/meme) to identify conserved motifs (Bailey et al., 2015), with the maximum number of motifs set to 10 and other parameters maintained as defaults. The 2000 bp upstream promoter sequences of *GmbZIP* genes were extracted using TBtools and analyzed for cis-acting regulatory elements via the PlantCARE database (http://bioinformatics.psb.ugent.be/webtools/plantcare/html/). Visualization of conserved domains, gene clusters, gene structures, motifs, and cis-elements was performed using TBtools.

### 2.4 Phylogenetic analysis and classification of GmbZIP gene family

To investigate the phylogenetic relationships between soybean and *Arabidopsis* bZIP proteins, we performed multiple sequence alignment of their protein sequences using MEGA 7.0 software, followed by phylogenetic tree construction with the maximum likelihood (ML) method. The resulting phylogenetic tree was visualized and beautified using Evolview (Subramanian et al., 2019) (https://evolgenius.info/evolview-v2/#login). Based on the established classification system for *Arabidopsis* bZIP gene families (Dröge-Laser et al., 2018), the GmbZIP gene family was categorized into 13 distinct subfamilies.

### 2.5 KEGG pathway and expression pattern analysis of *GmbZIP* genes

Using the BLAST alignment tool, the sequences of soybean *GmbZIP* genes were aligned against the KEGG public database (https://www.kegg.jp/) to obtain their functional annotations. The annotated information was then submitted to the BMKCloud online analysis platform (https://international.biocloud.net/zh/software/tools/) for visualization. Relative expression levels of genes were standardized by fragments per kilobase of transcript per million fragments mapped (FPKM) (Ma et al., 2024). The FPKM values of *GmbZIP* genes under different stress treatment groups were analyzed to investigate their expression patterns.

### 2.6 Analysis of collinearity and duplication events in the GmbZIP gene family

The ‘one step MCScanX’ function in TBtools was used to analyze the interspecific collinearity between soybean and *M. truncatula*, *Arabidopsis*, *S. lycopersicum*, *O. sativa* and *Z. mays*, as well as the intraspecific collinearity of soybean. The results were visualized using ‘Dual collinearity Plot for MCScanX’ and ‘Advanced Circo’ functions in TBtools for interspecies and intraspecies collinearity, respectively. The evolutionary relationships between genes were assessed by calculating the synonymous substitution rate (Ks) and nonsynonymous substitution rate (Ka). The Ka/Ks ratio for syntenic *GmbZIP* gene pairs in soybean was computed using the ‘Simple Ka/Ks Calculator’ tool in TBtools. All duplicated gene pairs of *GmbZIP* genes in soybean were classified into the following four categories: segmental, tandem, proximal, and dispersed duplications. The divergence time (T, million years ago, MYA) of gene duplication was estimated using the formula: T = Ks/2λ, where λ is 1.5 × 10 ^− 8^.

### 2.7 Mining of candidate *GmbZIP* genes for salt stress in soybean

By reviewing the literature, we retrieved bZIP genes associated with salt stress in the model plant *Arabidopsis* and downloaded their protein sequences. These sequences were then aligned with soybean *GmbZIP* genes for homology analysis. Candidate genes for salt stress response in soybean were screened based on: homologous gene alignment, functional annotation, KEGG enrichment analysis, Protein-protein interaction (PPI) network prediction. This multi-step approach allowed us to identify potential *GmbZIP* genes involved in soybean salt stress tolerance.

### 2.8 Protein-protein interaction network prediction

To better understand the interactions between GmbZIP proteins and other proteins, and to predict the functions of salt stress-responsive candidate *GmbZIP*, we constructed PPI networks for the candidate GmbZIP proteins using the STRING database (https://cn.string-db.org/). After inputting the protein sequences into the database, interaction networks with other proteins were generated. The strength of protein-protein interactions in the network was then calculated using the K-means clustering algorithm (Ahmad et al., 2020).

### 2.9 RNA extraction, cDNA synthesis, and qRT-PCR analysis

The total RNA of the samples was extracted using the Promega total RNA extraction kit, and the concentration and purity of the obtained RNA samples were detected on the NanoDrop 2000/2000C spectrophotometer (Thermo Scientific, USA). Finally, the reverse transcription kit provided by Vazyme Company was used to synthesize high-quality cDNA. Quantitative real-time PCR (qRT-PCR) primers of GmbZIP genes were designed by Premier 6.0 software with GmACTIN as the internal reference gene (**Supplementary Table 1**). The qRT-PCR was performed using a special 384-well PCR plate and 10 μL system (SYBR^®^ Green Realtime PCR Master Mix: 5.0 μL; Forward primer: 0.5 μL; Reverse primer: 0.5 μL; cDNA template: 1.0 μL; ddH_2_O: 3.0 μL). The relative expression of the gene was analyzed according to the 2 ^− ΔΔCT^ method (Cao et al., 2019) and the significance analysis and plotting were performed by GraphPad Prism 8.

## 3 Results

### 3.1 Identification and physicochemical property analysis of *GmbZIP* gene in soybean

Based on the soybean whole-genome data, 236 candidate *GmbZIP* genes were initially identified, corresponding to a total of 495 protein sequences. After removing erroneous and redundant sequences, 171 *GmbZIP* genes comprising 385 protein sequences were ultimately identified and renamed as *GmbZIP1* to *GmbZIP171*. Among the 385 GmbZIP protein sequences, GmbZIP139–1 encodes the smallest number of amino acids (106 aa), while GmbZIP26–1 encodes the largest (853 aa). The molecular weights ranged from 12.10 to 94.10 KDa. The pI varied between 4.65 and 10.63, with 233 proteins having a pI < 7 (acidic proteins) and 152 proteins having a pI > 7 (basic proteins). The instability index ranged from 35.34 to 83.01, with 8 proteins exhibiting an index < 40 (stable proteins) and 377 proteins showing an index > 40 (unstable proteins). The aliphatic index ranged from 45.97 to 103.11. And the GRAVY ranged from -1.12 to -0.08, all of which were below 0, indicating that all 385 GmbZIP proteins are hydrophilic. Subcellular localization predictions revealed that 169 *GmbZIP* genes are localized in the nucleus, while only *GmbZIP54* and *GmbZIP134* are localized in the chloroplast (**Supplementary Table 2**). Additionally, we observed the loss of the bZIP domain in 13 proteins from 7 *GmbZIP* genes, namely *GmbZIP12*, *GmbZIP20*, *GmbZIP26*, *GmbZIP35*, *GmbZIP120*, *GmbZIP139*, and *GmbZIP169*. These findings provide a theoretical foundation for further studies on the purification, activity, and function of GmbZIP proteins.

### 3.2 Identification and expression pattern analysis of stress-responsive *GmbZIP* genes

**Figure 1:**
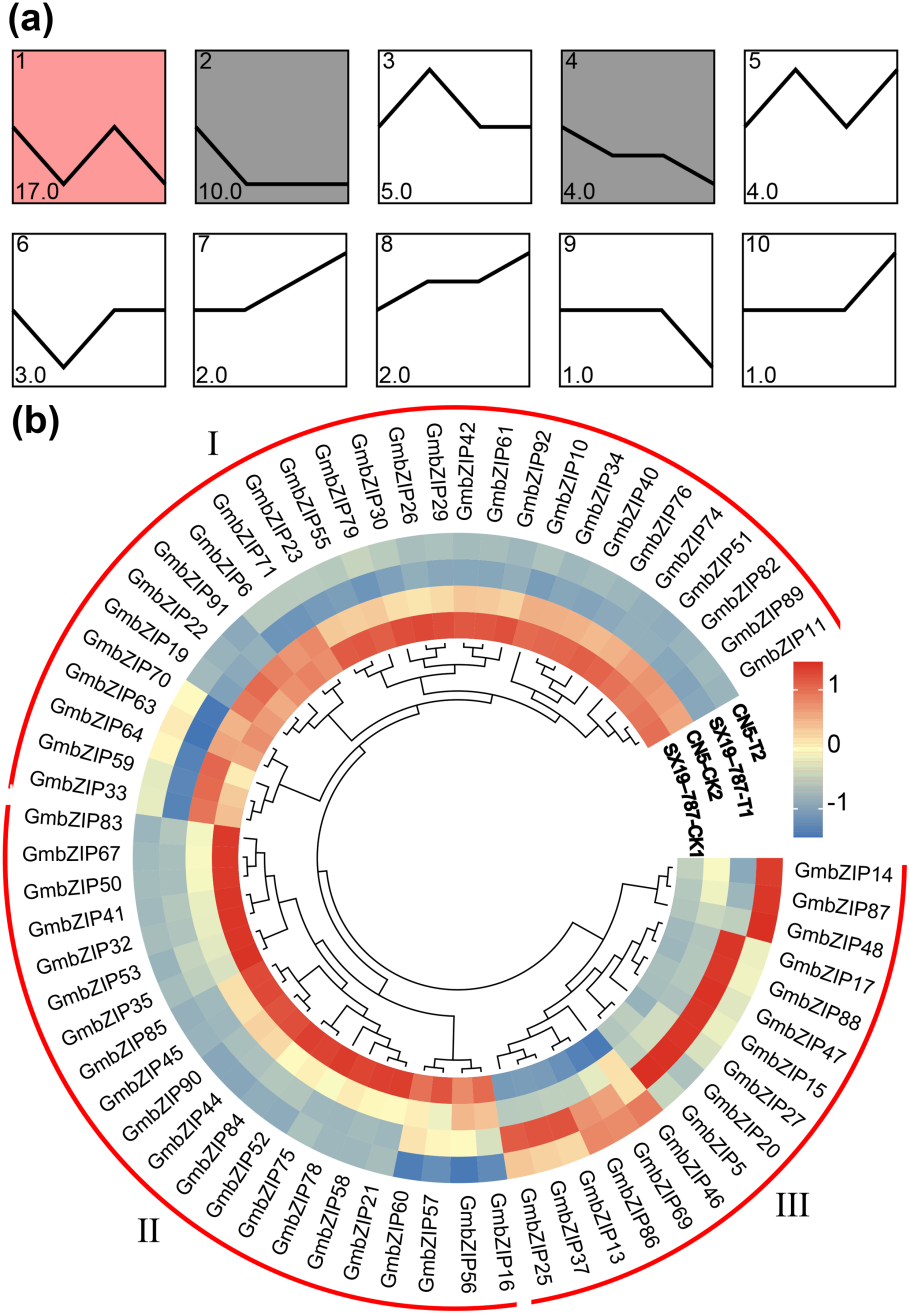
Analysis of expression trends and patterns of differentially expressed *GmbZIP* genes under waterlogging stress. **(a)** Expression trend analysis of *GmbZIP* genes. **(b)** Expression pattern analysis of *GmbZIP* genes, where CK represents the control group and T represents the waterlogging treatment group.

In this study, we identified 65 differentially expressed *GmbZIP* genes from the transcriptome data of soybean seeds under waterlogging stress. Trend analysis revealed that 49 of these *GmbZIP* genes (16 excluded due to fold-change < 2) were primarily distributed across 10 expression trend profiles. Among them, profile 1 contained the highest number of *GmbZIP* genes (17), followed by profile 2 (10). Further significance analysis using the Short Time-series Expression Miner (STEM) (Fan et al., 2021a) software identified three significant (P < 0.05) expression profiles (profile 1, profile 2, and profile 4), comprising a total of 31 *GmbZIP* genes (63.3%). Three significant profile mainly showed that the expression level of *GmbZIP* gene decreased under waterlogging stress (**Figure 1A**). Additionally, we performed expression pattern and cluster analysis on all 65 *GmbZIP* genes, categorizing them into three major groups: group I contained 28 *GmbZIP* genes, which were mainly highly expressed in the control group, and group II contained 21 *GmbZIP* genes, which were predominantly upregulated in SX19-787-CK, group III contained 16 *GmbZIP* genes, which were significantly induced in the waterlogging-stress treatment group (**Figure 1B**). This analysis elucidates the dynamic expression patterns of *GmbZIP* genes under flooding stress, providing critical insights into the molecular mechanisms underlying soybean flooding tolerance.

**Figure 2:**
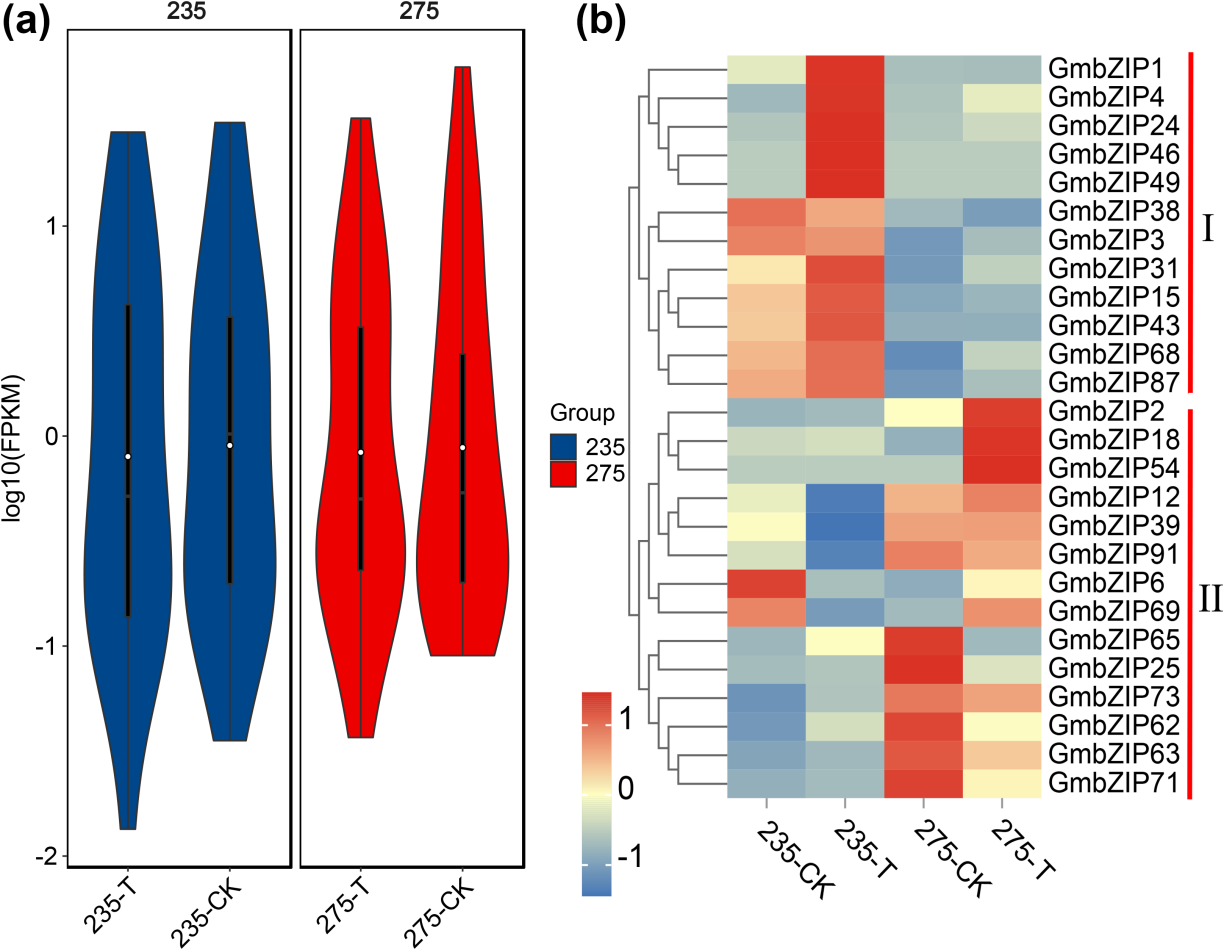
Analysis of expression pattern of *GmbZIP* gene related to shading stress. **(a)** The violin diagram shows the distribution of *GmbZIP* expression level. **(b)***GmbZIP* expression pattern analysis, where CK represents the control group and T represents the shading treatment group.

Based on the crucial roles of bZIP genes in plant growth regulation and light signal transduction (Jiang et al., 2023), this study conducted shade stress treatments on shade-tolerant material 275 and shade-sensitive material 235. The results demonstrated that 26 *GmbZIP* genes responded to shade stress, exhibiting differential expression patterns. Notably, in shade-tolerant material 275, shade treatment induced an overall downregulation trend in these genes, which may represent an adaptive mechanism to maintain internal homeostasis by reducing the expression of certain genes (such as reducing energy consumption). Further analysis revealed that compared to material 235, shade-tolerant material 275 showed significantly reduced gene expression dispersion (more concentrated distribution) under shade treatment, suggesting that the shade-tolerant material may employ more precise transcriptional regulation to avoid drastic fluctuations in gene expression, thereby better adapting to shade stress conditions (**Figure 2A**). Through systematic cluster analysis, we categorized the 26 *GmbZIP* genes into 2 major groups and 4 subfamilies. Among them, genes such as *GmbZIP2*, *GmbZIP18*, and *GmbZIP54* showed significant upregulation under shade stress, indicating their potential direct involvement in the regulation of plant responses to shade stress(**Figure 2B**).

**Figure 3:**
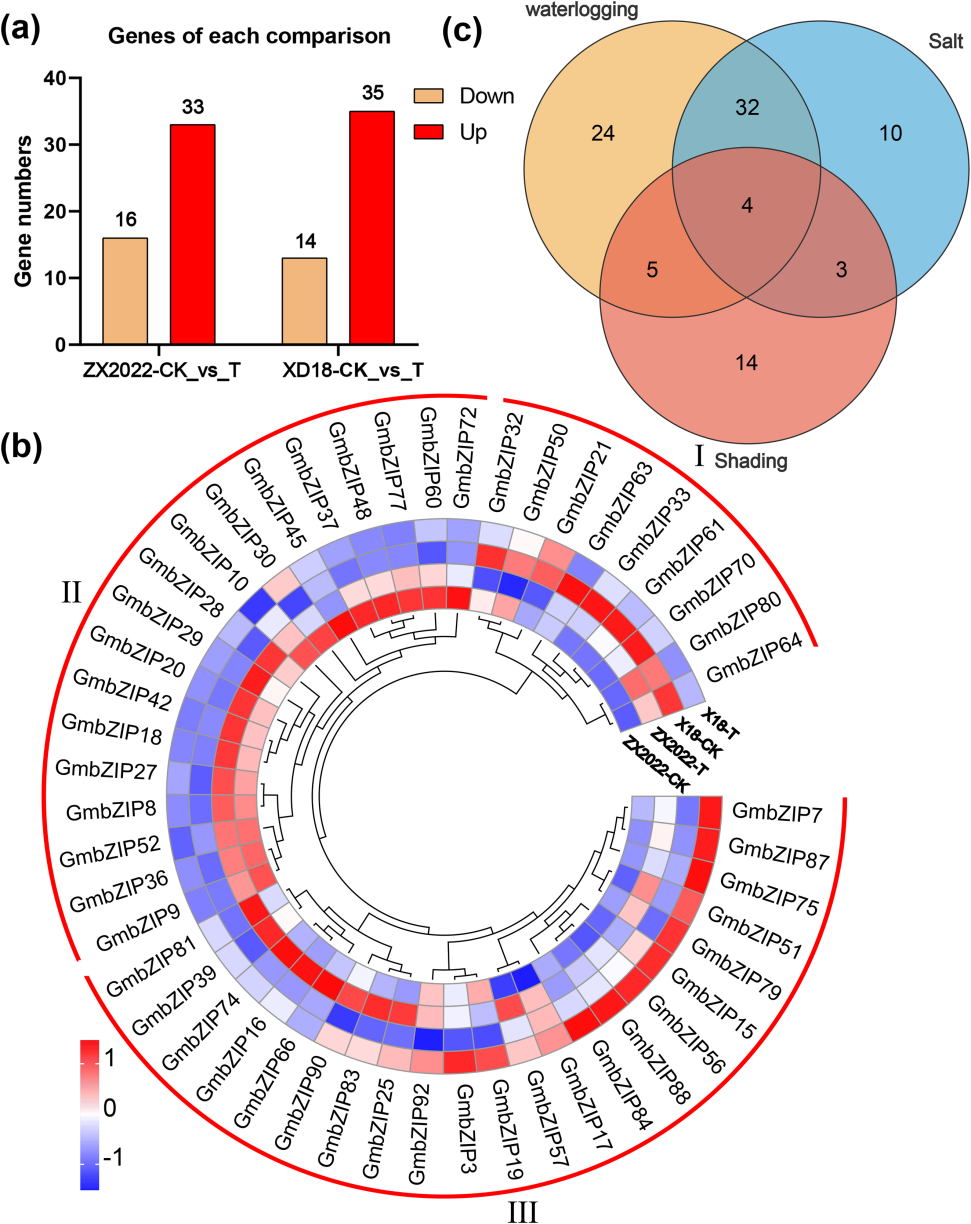
Analysis of differentially expressed *GmbZIP* gene expression patterns under salt stress. **(a)** The distribution of *GmbZIP* gene in the comparison group. **(b)** Analysis of *GmbZIP* gene expression pattern. **(c)** Venn diagram showed the distribution of *GmbZIP* genes in stress treatment (flooding, shading and salt).

To investigate the regulatory function of the *GmbZIP* gene in soybean salt stress tolerance, we systematically identified 49 differentially expressed *GmbZIP* genes using soybean salt stress transcriptome data. Among them, salt stress treatment induced the upregulation of 33 *GmbZIP* genes in the salt-sensitive material ZX2022, and 35 *GmbZIP* genes showed elevated expression levels in the salt-tolerant material XD18 (**Figure 3A**). Through further expression pattern and cluster analysis, 49 *GmbZIP* genes were divided into 3 groups (with five sub-branches). Group I contained nine *GmbZIP* genes, which were highly expressed mainly in XD18-CK. Group II comprised 18 *GmbZIP* genes distributed across two sub-branches, showing high expression in ZX2022-CK and ZX2022-T, respectively. Group III included 22 *GmbZIP* genes, which were significantly upregulated under salt stress treatment (**Figure 3B**).

By integrating transcriptome data from waterlogging, shading, and salt stress, a total of 92 differentially expressed *GmbZIP* genes were identified. Venn diagram analysis revealed that 36 genes responded to both waterlogging and salt stress, nine genes were differentially expressed under both waterlogging and shading stress, and seven genes exhibited expression changes under both salt and shading stress. Notably, four core genes (*GmbZIP15*, *GmbZIP25*, *GmbZIP63* and *GmbZIP87*) were significantly differentially expressed under all three stress treatments. These results suggest that some *GmbZIP* genes may participate in the coordinated regulation of soybean responses to multiple abiotic stresses. In particular, *GmbZIP15*, *GmbZIP25*, *GmbZIP63* and *GmbZIP87* may serve as key regulatory factors, broadly involved in soybean stress response mechanisms (**Figure 3C**).

### 3.3 KEGG enrichment analysis of *GmbZIP* gene

**Figure 4:**
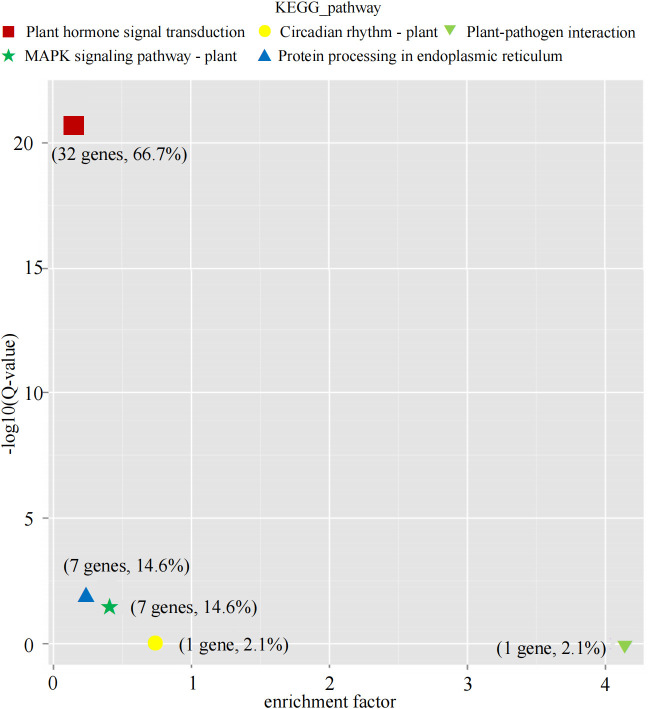
KEGG enrichment analysis of stress-related *GmbZIP* gene.

To gain deeper insights into the regulatory roles of *GmbZIP* genes in abiotic stress responses, we performed KEGG pathway enrichment analysis on the 92 abiotic stress-responsive *GmbZIP* genes. The results revealed that 48 *GmbZIP* genes were significantly enriched in five key pathways: “Plant hormone signal transduction”, “MAPK signaling pathway-plant”, “Protein processing in endoplasmic reticulum”, “Circadian rhythm-plant” and “Plant-pathogen interaction”. Notably, the “Plant hormone signal transduction” pathway exhibited the highest enrichment significance and contained the largest number of *GmbZIP* genes (32 genes, 66.7%, **Figure 4**).

### 3.4 Phylogenetic analysis of *GmbZIP* gene

**Figure 5:**
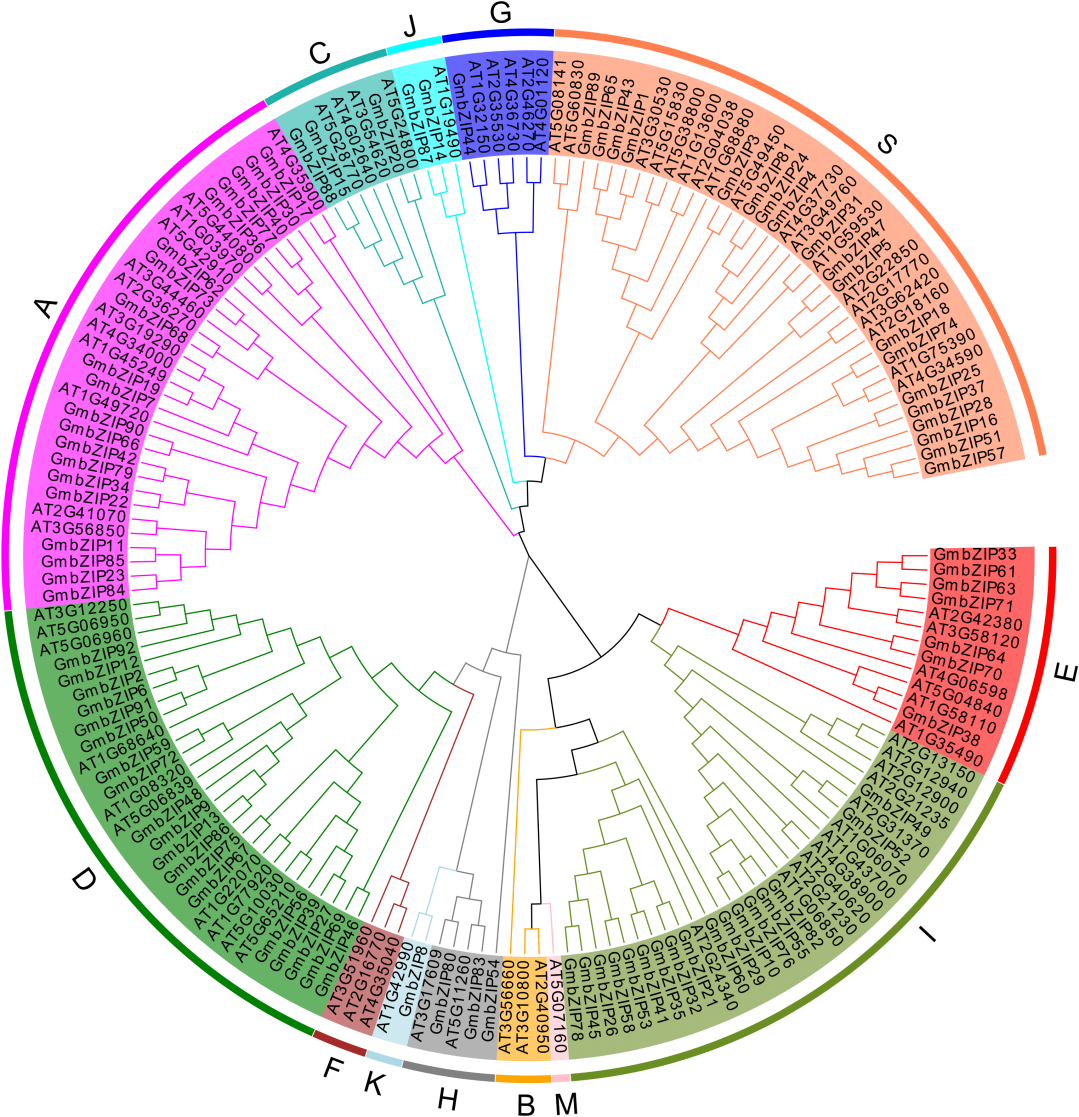
Phylogenetic tree of bZIP gene in soybean and *Arabidopsis*. The capital letters denote different subfamilies.

Phylogenetic analysis, as an effective approach for elucidating the evolutionary history, origin, and functional divergence of homologous gene families across species, was employed in this study to investigate the evolutionary relationships of the soybean GmbZIP gene family. Based on multiple sequence alignments of 92 soybean *GmbZIP* genes and 75 *Arabidopsis AtbZIP* genes, we constructed a phylogenetic tree and classified the GmbZIP family members into 13 subfamilies (A, B, C, D, E, F, G, H, I, J, K, M, and S) according to the *Arabidopsis AtbZIP* classification system. Phylogenetic analysis revealed significant variations in member distribution among subfamilies (ranging from 1 to 20). The A subfamily contained the highest number of members (20 *GmbZIPs*), followed by the D and S subfamilies (19 each), whereas the K and G subfamilies had only a single member. Notably, the B, F, and M subfamilies, which exist in *Arabidopsis*, were absent from the identified set of soybean stress-responsive *GmbZIP* genes. Furthermore, the phylogenetic tree demonstrated that some *GmbZIP* genes clustered closely with *Arabidopsis AtbZIP* genes (**Figure 5**), suggesting that they may encode functionally conserved homologous proteins and retain similar biological functions.

### 3.5 Gene structure, conserved domain and motif analysis of *GmbZIPs*

**Figure 6:**
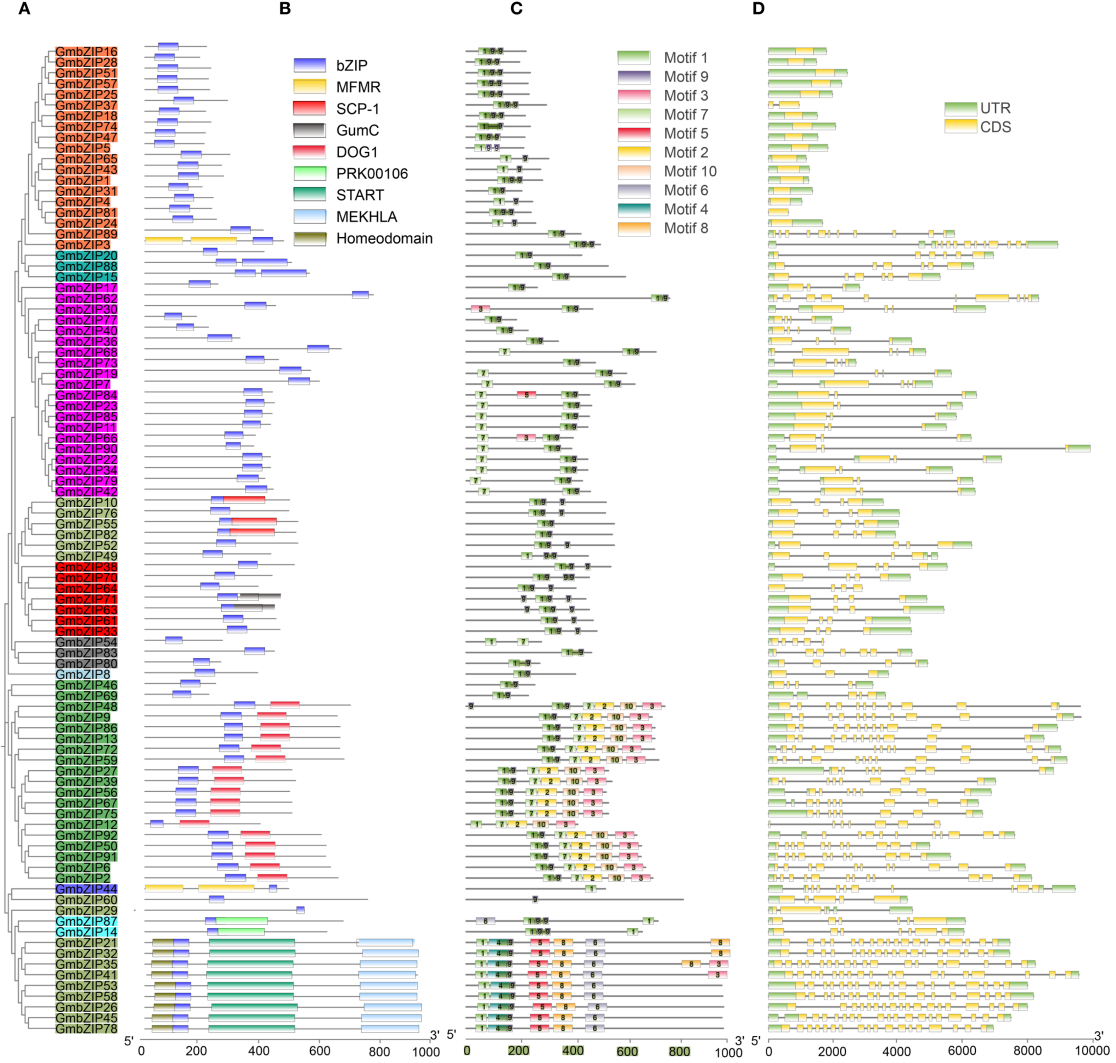
Gene structure, conserved domain and motif distribution of *GmbZIPs*. **(A)** Phylogenetic tree of *GmbZIPs*. **(B)** Conserved domain distribution of *GmbZIPs*. **(C)** Conserved motif distribution of *GmbZIPs*. **(D)***GmbZIPs* gene structure characteristics.

The distribution characteristics of conserved domains within a gene family can effectively reflect the evolutionary relationships and functional properties of genes. Among the 92 soybean *GmbZIP* transcription factors, A total of nine types of conserved domains were identified, including bZIP, MFMR, SCP-1, GumC, DOG1, PRK00106, START, MEKHLA, and Homeodomain. Phylogenetic analysis revealed that members of subfamilies A, C, H, K, and S (except *GmbZIP3*) only carry the core bZIP domain, with genes from each subfamily clustering into distinct branches (**Figure 6A**). Notably, other subfamilies exhibited significant domain diversity: subfamily I displayed the most complex domain composition (containing SCP-1, START, MEKHLA, and Homeodomain), while in subfamily D, all members except *GmbZIP46* and *GmbZIP69* retained both the bZIP and DOG1 domains (**Figure 6B**).

To investigate the motif characteristics of the 92 GmbZIP proteins, we identified 10 conserved motifs (Motif 1-10) using the MEME tool, revealing significant quantitative variation (1–7 motifs) among *GmbZIP* genes. Among these, Motif 1 was nearly universally distributed (absent only in *GmbZIP29*) and formed a stable tandem module with Motif 9. Sequence alignment confirmed that Motif 1 and Motif 9 correspond to the DNA-binding basic region and the leucine zipper region of the bZIP domain, respectively (**Supplementary Figure 1**). The motif distribution exhibited distinct subfamily-specific patterns: subfamily D was specifically enriched with Motif 2 and Motif 10, whereas a specific branch of subfamily I exclusively contained Motif 4, Motif 5, Motif 6, and Motif 8 (**Figure 6C**). This distribution pattern strongly correlated with phylogenetic relationships, as members of the same subfamily typically maintained similar motif compositions.

Analysis of the exon-intron organization in *GmbZIP* genes revealed a broad variation in exon numbers (ranging from 1 to 19, with *GmbZIP45* containing the most exons). Frequency distribution analysis showed that the 4-exon structure was the most common (21 genes), predominantly clustered in subfamily A. Notably, exon numbers exhibited a strong correlation with phylogenetic branching: subfamily S generally lacked introns, a specific branch of subfamily I consistently contained 18 or more exons, other subfamilies or branches typically had 3–6 exons (**Figure 6D**; **Supplementary Table 3**). This structural conservation further validated the classification system based on sequence similarity, indicating that members of the same subfamily share common evolutionary features in genetic composition and gene architecture.

### 3.6 Cis-acting elements analysis in *GmbZIPs* promoter regions

**Figure 7:**
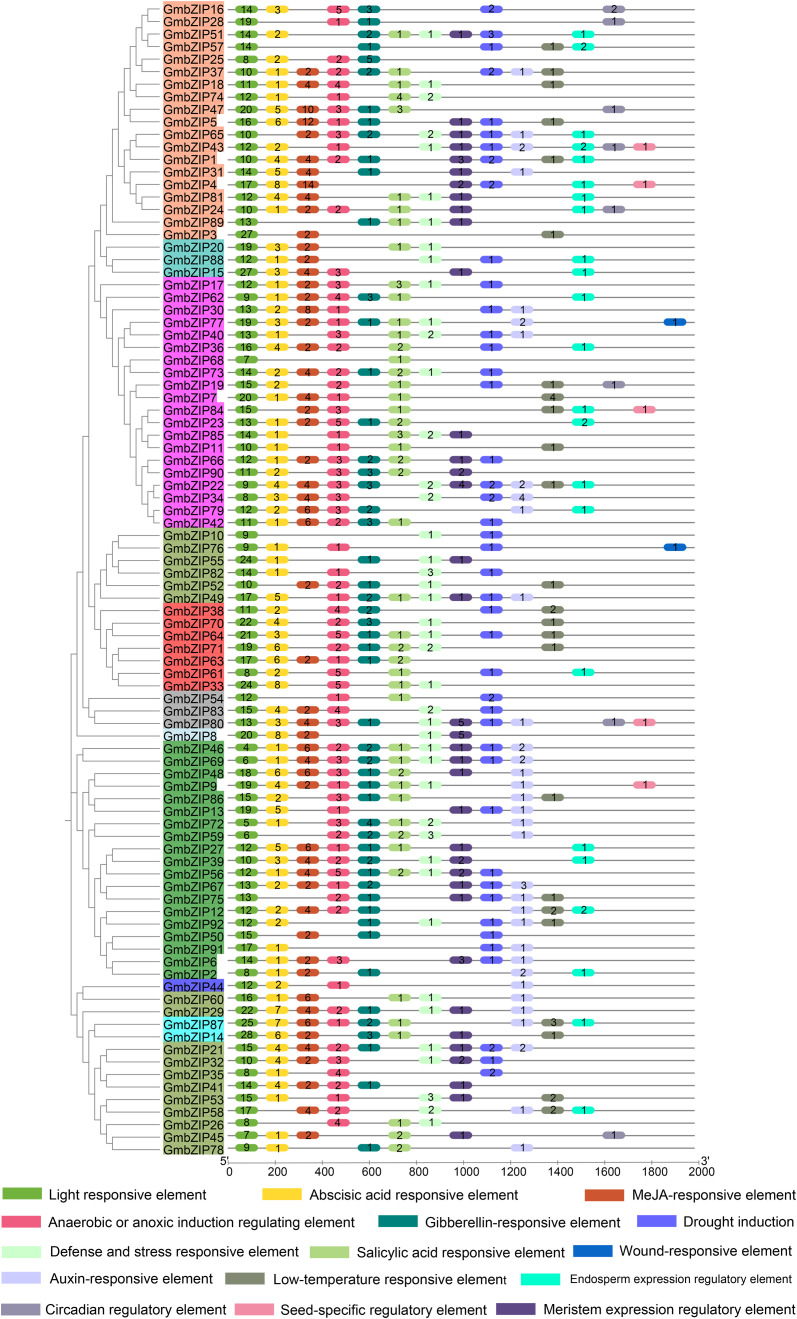
Analysis of cis-acting elements of *GmbZIPs*. Numbers indicate the number of cis-acting elements.

Systematic analysis of promoter cis-acting elements represents a crucial approach to elucidating gene function and tissue-specific expression patterns (Schmitz et al., 2022). To investigate the regulatory mechanisms of the GmbZIP gene family in stress responses, this study predicted cis-elements within the 2.0 kb upstream promoter region of *GmbZIP* genes using the PlantCARE database. Bioinformatics analysis identified a total of 2,311 cis-regulatory elements in the *GmbZIP* promoter regions, which were classified into four functional categories (**Figure 7**; **Supplementary Table 4**): 1. Light-responsive elements (including core motifs such as GT1-motif, G-box, Box 4, and TCT-motif); 2. Growth and development-related elements (encompassing regulatory elements for meristem expression, endosperm expression, circadian rhythm regulation, and seed-specific regulation); 3. Biotic and abiotic stress-responsive elements (involving anaerobic/anoxic induction, defense and stress response, drought induction, low-temperature response, and wound response elements); 4. Phytohormone-responsive elements, including abscisic acid (ABA), gibberellin, auxin, methyl jasmonate (MeJA), and salicylic acid (SA) response elements. Notably, light-responsive elements were detected in all *GmbZIP* transcription factors, though their numbers varied significantly, ranging from 4 (*GmbZIP46*) to 28 (*GmbZIP14*). Phytohormone-responsive elements were widely distributed, with ABA-responsive elements (present in 77 *GmbZIPs*) and MeJA-responsive elements (found in 54 *GmbZIPs*) being the most abundant. Strikingly, *GmbZIP4* harbored an exceptionally high number (14) of MeJA-responsive elements. Additionally, stress-responsive elements were identified in 87 *GmbZIP* genes, whereas growth and development-related elements exhibited the lowest distribution frequency. These findings indicate that the 92 *GmbZIP* genes may coordinately regulate the spatiotemporal expression patterns of downstream responsive genes under stress conditions via the enriched light-responsive, phytohormone-responsive and stress-responsive cis-acting elements in their promoter regions, thereby modulating plant adaptability to adverse environmental conditions.

### 3.7 Chromosomal distribution and collinearity analysis of *GmbZIP* genes

**Figure 8:**
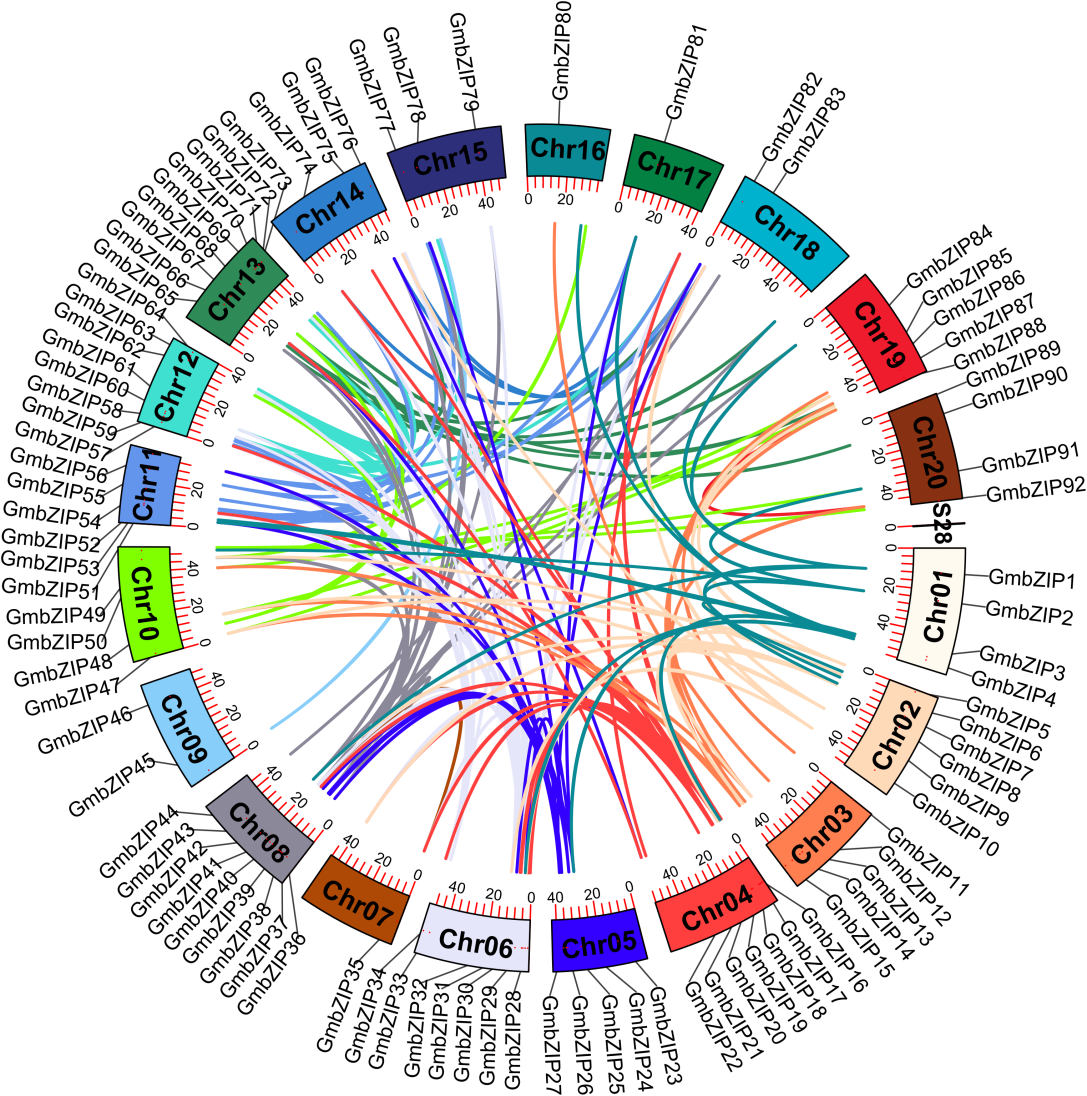
Chromosome distribution and intraspecific synteny analysis of *GmbZIP* gene. Colored rectangles represent 20 chromosomes, and lines of different colors represent syntenic gene pairs.

Based on the soybean genome data, 92 *GmbZIP* genes were physically mapped to 20 chromosomes and showed a significantly uneven distribution. Among them, chromosomes 8 and 13 contained the highest number of genes (9 each), followed by chromosome 12 (8 genes). In contrast, chromosomes 7, 16, and 17 each contained only one *GmbZIP* gene (**Figure 8**).

Gene duplication events (including tandem and segmental duplications) play a crucial role in the expansion and functional diversification of gene families (Fan et al., 2021b). In this study, a total of 175 duplication events were detected among the 92 stress-responsive *GmbZIP genes*, with segmental duplications being predominant (173 pairs), while only two tandem duplication events were identified (*GmbZIP41*-*GmbZIP116* and *GmbZIP54*-*GmbZIP128*). This indicates that segmental duplication is the primary mechanism for the expansion of this gene family (**Figure 8**; **Supplementary Table 5**). Additionally, four *GmbZIP* genes exhibited syntenic relationships with genes non-family genes, such as *GmbZIP10* and *Glyma.06G143200*, suggesting that these genes may participate in soybean stress response through coordinated regulation. Expression analysis revealed that some gene pairs (e.g., *GmbZIP1* and *GmbZIP146*) may have undergone functional divergence during evolution, which specifically showed that *GmbZIP146* exhibited no differential expression under stress conditions. To further trace the evolutionary history of the *GmbZIP* genes, the divergence time of gene pairs was estimated based on the synonymous substitution rate (Ks). The results showed that the divergence times of all gene pairs spanned a wide range, from 4.76 to 343.34 million years ago (Mya). The earliest divergence occurred between *GmbZIP28* and *GmbZIP74* (343.34 Mya). To analyze the selective pressures during evolution, the nonsynonymous substitution rate (Ka) and Ka/Ks ratios were calculated. All gene pairs exhibited Ka/Ks values less than 1, indicating that the *GmbZIP* genes underwent widespread purifying selection during their evolution from 4.76 to 343.34 Mya, suggesting that their function was strongly constrained (**Supplementary Table 5**).

**Figure 9:**
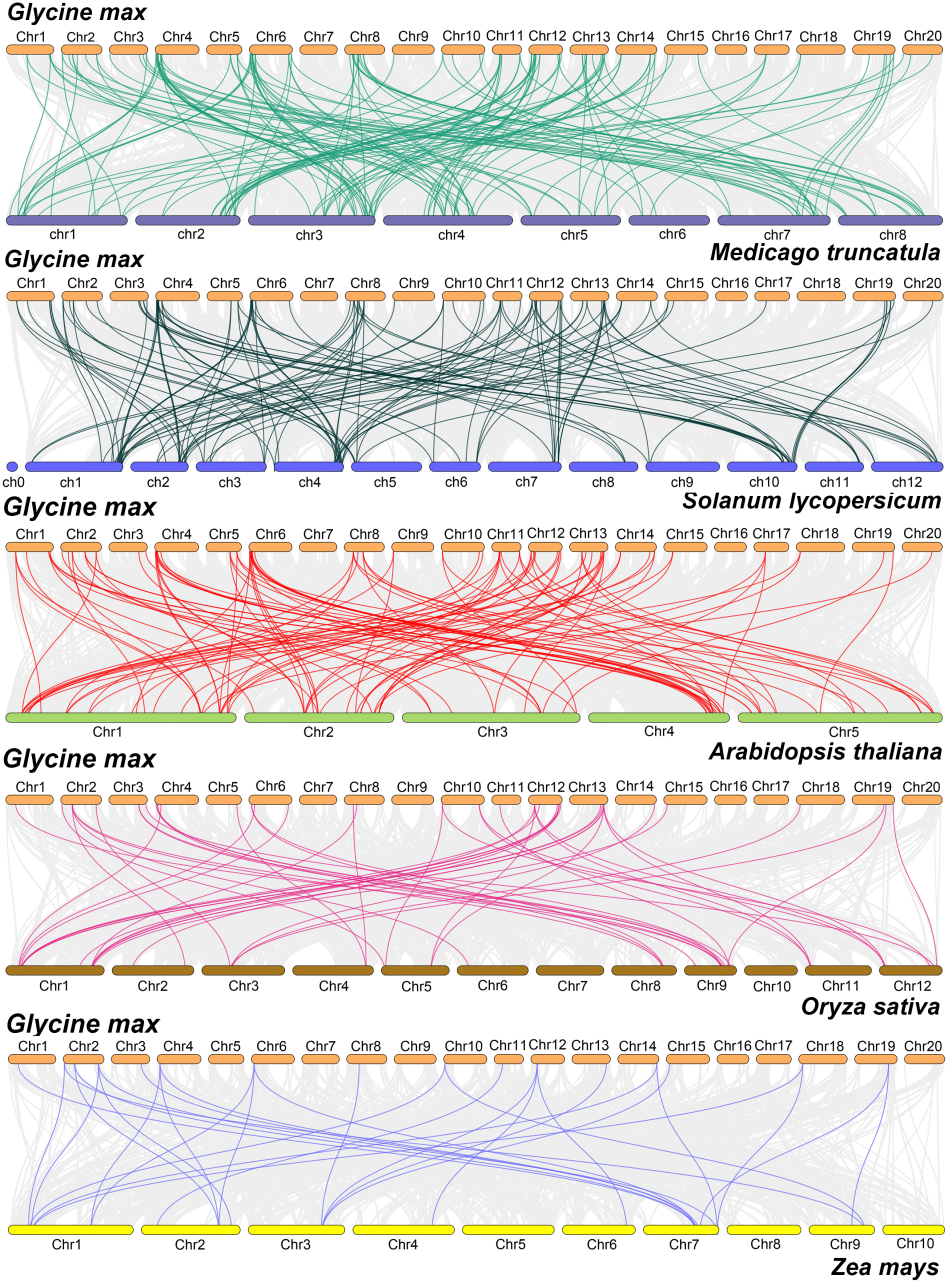
Collinearity analysis of *bZIP* genes between soybean and five other representative plant species. lines of different colors represent collinear gene pairs.

To further investigate the evolutionary relationships of the bZIP gene family across diverse species, we constructed collinearity maps between soybean and three dicotyledonous species (*M. truncatula*, *Arabidopsis*, and *S. lycopersicum*) as well as two monocotyledonous species (*O. sativa* and *Z. mays*). The analysis revealed that the largest number of collinear gene pairs was between soybean and *M. truncatula* (143 pairs), followed by *S. lycopersicum* (122 pairs), *Arabidopsis* (104 pairs), *O. sativa* (49 pairs), and *Z. mays* (30 pairs). Notably, 58 *GmbZIP* genes exhibited at least two pairs of collinear relationships with other species. Among them, 11 genes even formed four collinear pairs, suggesting that these genes may play crucial and conserved roles in the evolution of the bZIP family. Overall, the number of collinear bZIP gene pairs between soybean and dicotyledonous plants was significantly higher than that between soybean and monocotyledonous plants. This result supports a closer phylogenetic relationship between soybean and dicotyledons, while also reflecting the higher conservation of *bZIP* genes during the evolution of dicotyledonous plants. Additionally, collinearity analysis identified 12 highly conserved *GmbZIP* genes, including *GmbZIP5*, *GmbZIP7*, *GmbZIP10*, *GmbZIP13*, *GmbZIP19*, *GmbZIP30*, *GmbZIP47*, *GmbZIP52*, *GmbZIP59*, *GmbZIP72*, *GmbZIP76*, and *GmbZIP82*. These genes showed collinear relationships across all compared species, indicating that they might have originated from the common ancestral genome prior to the divergence of monocotyledonous and dicotyledonous plants. At the subfamily level, the collinear patterns of *GmbZIP* genes also exhibited certain evolutionarily conserved characteristics. Notably, no collinear genes were detected for members of subfamily H across all compared species, implying that this subfamily might have undergone frequent genomic rearrangements or gene loss events following species divergence. In contrast, multiple *GmbZIP* genes in subfamily S exhibited significant collinear associations with the identical *bZIP* gene in *M. truncatula*, *S. lycopersicum*, and *Arabidopsis* (**Figure 9**; **Supplementary Table 6**).

### 3.8 Mining and verification of candidate *GmbZIP* genes under salt stress

This study employed a comprehensive suite of bioinformatic approaches, including phylogenetic tree construction, genomic collinearity analysis, and BLAST homology alignment, to systematically identify salt stress-responsive *GmbZIP* genes in the soybean genome. Initially, potential homologous genes were preliminarily screened based on the phylogenetic relationships and genomic collinearity analysis of bZIP proteins in soybean and *Arabidopsis*. Subsequently, homologous relationships between soybean and *Arabidopsis bZIP* genes were further verified through BLASTP alignment and functional annotation data. Ultimately, by integrating existing literature evidence, 12 *GmbZIP* genes potentially involved in salt stress response were selected (**Table 1**). Among these candidate genes, *GmbZIP7*, *GmbZIP19*, and *GmbZIP30* are all members of the ABF (ABA RESPONSIVE ELEMENT BINDING FACTOR) family. Research by Du et al., demonstrated that *ABF1*, *ABF2*, *ABF3*, and *ABF4* in *Arabidopsis* enhance plant salt stress tolerance (Du et al., 2023). *GmbZIP45* is homologous to *Arabidopsis AT2G34710* (*PHB*, PHABULOSA). Studies have shown that under salt stress, miR165 and miR166 co-regulate the development of root plasticity by modulating the expression of PHB protein, and PHB mutants exhibit enhanced resistance to salt stress (Scintu et al., 2023). *GmbZIP52* shows a collinear relationship with *Arabidopsis AT2G31370* (*POSF21*) and shares consistent functional annotation. Van Oosten et al., found that the *Arabidopsis POSF21* mutant air1 fails to accumulate anthocyanin normally under salt stress conditions (Van Oosten et al., 2013). TGA (TGACG motif-binding factor) transcription factors belong to subfamily D of the bZIP gene family. Previous studies have reported that the overexpression of *GmTGA13* (*Glyma.10G296200*) and *GmTGA17* (*Glyma.12G184500*) significantly improves soybean salt-stress tolerance (Li et al., 2019; Ke et al., 2022). This study identified another TGA family member, *GmbZIP72* (*TGA9*), which is potentially involved in salt stress response. Overexpression of *PpTGA9* has been confirmed to enhance salt stress resistance in *Arabidopsis* (Wu et al., 2023a). *GmbZIP83* is homologous to *Arabidopsis AT5G11260* (*HY5*, ELONGATED HYPOCOTYL 5), a gene primarily involved in light signal transduction. Research by Yang et al., demonstrated that *HY5* deficiency leads to significantly enhanced salt sensitivity in *Arabidopsis* (Yang et al., 2023a). DC3 Promoter Binding Factors (DPBFs) belong to subfamily A of the bZIP family and have been reported to participate in stress-response regulation (Qian et al., 2019). In this study, *GmbZIP17*, *GmbZIP42*, *GmbZIP79*, *GmbZIP84*, and *GmbZIP90* were all annotated as DPBF3. In *Arabidopsis*, the *AtDPBF3* mutant exhibits increased sensitivity to salt stress, indicating that this gene plays a positive role in enhancing salt stress tolerance (Sun et al., 2025).

**Table 1:** Orthologous *bZIP* genes in soybean and *Arabidopsis*.

Candidate gene	Function	Annotate	*Arabidopsis*	Reference
*GmbZIP7*	Salt-tolerant gene	ABF4 or ABF1	*AT3G19290* or *AT1G49720*	(Du et al., 2023)
*GmbZIP19*	Salt-tolerant gene	ABF2	*AT1G45249*	(Zhang et al., 2024)
*GmbZIP30*	Salt-tolerant gene	ABF2	*AT1G45249*	(Zhang et al., 2024)
*GmbZIP45*	Regulation of root plastic development under salt stress	PHB	*AT2G34710*	(Scintu et al., 2023)
*GmbZIP52*	Regulation of anthocyanin accumulation under salt stress	POSF21	*AT2G31370*	(Van Oosten et al., 2013)
*GmbZIP72*	Salt-tolerant gene	TGA9	*AT1G08320*	(Wu et al., 2023a)
*GmbZIP83*	Salt-tolerant gene	HY5	*AT5G11260*	(Yang et al., 2023a)
*GmbZIP17*	Salt-tolerant gene	DPBF3	*AT3G56850*	(Sun et al., 2025)
*GmbZIP42*	Salt-tolerant gene	DPBF3	*AT3G56850*	(Sun et al., 2025)
*GmbZIP79*	Salt-tolerant gene	DPBF3	*AT3G56850*	(Sun et al., 2025)
*GmbZIP84*	Salt-tolerant gene	DPBF3	*AT3G56850*	(Sun et al., 2025)
*GmbZIP90*	Salt-tolerant gene	DPBF3	*AT3G56850*	(Sun et al., 2025)

**Figure 10:**
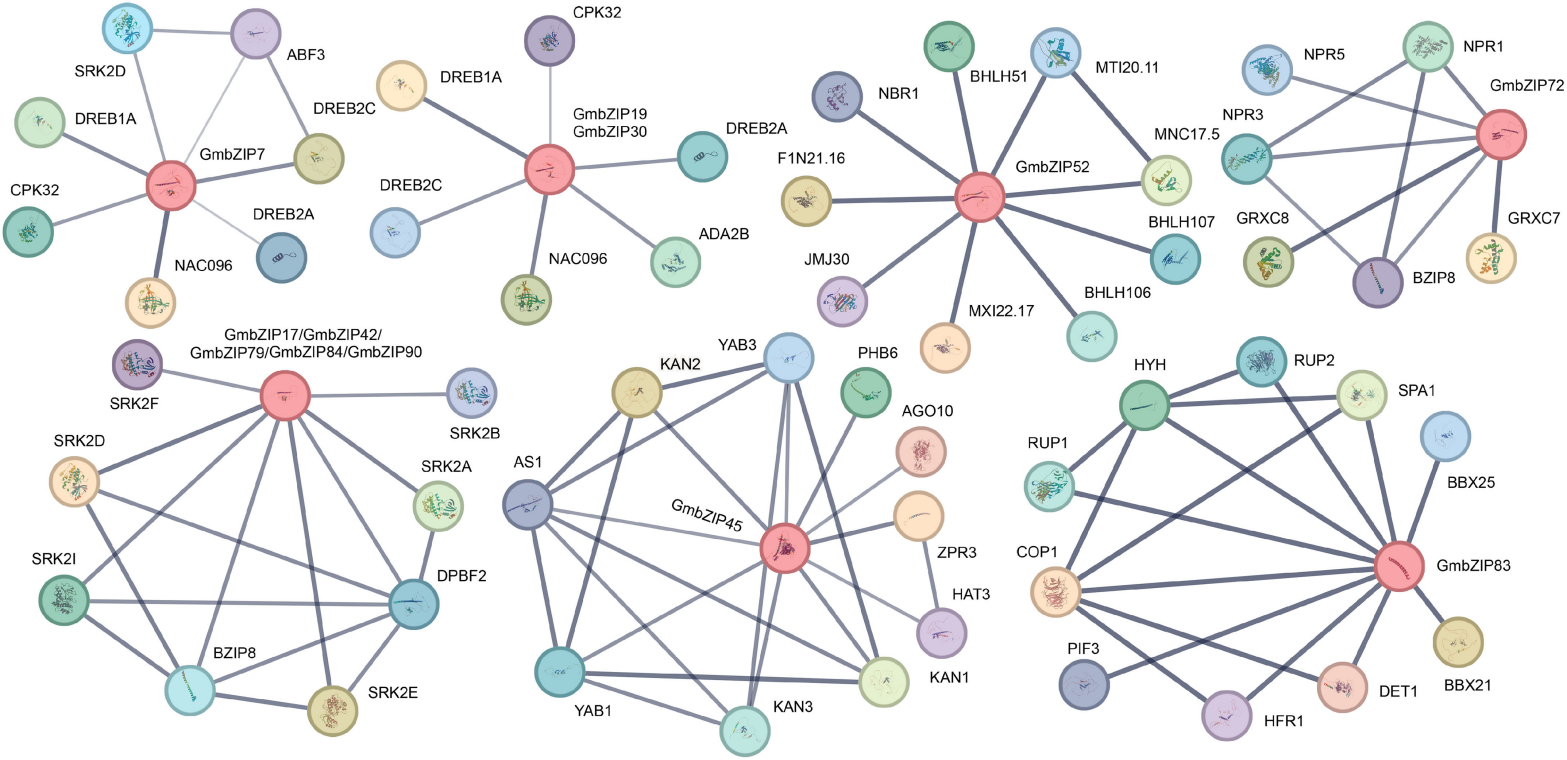
Protein interaction network analysis of salt stress candidate *GmbZIP* gene.

To deeply explore the regulatory networks and functions of these 12 salt stress-responsive candidate genes, it is particularly crucial to elucidate the potential interactions among their encoded proteins. Therefore, this study constructed a protein-protein interaction (PPI) network map for these candidate genes (**Figure 10**). The results revealed that *GmbZIP7*, *GmbZIP19*, and *GmbZIP30* primarily interact with members of the DREB (Dehydration-Responsive Element Binding) family, such as *DREB1A*, *DREB2A*, and *DREB2C*. These DREB factors have been reported to enhance plant salt stress tolerance (Shafeinie et al., 2014; Song et al., 2014; Alvarez-Gerding et al., 2015; Yan et al., 2023). *GmbZIP45* exhibited the most significant interactions with *KANADI* (*KAN*) genes, including *KAN1*, *KAN2*, and *KAN3*. *GmbZIP52* strongly interacted with members of the bHLH family, namely *bHLH51*, *bHLH106*, and *bHLH107*. Notably, *bHLH106* is also involved in salt stress regulation, and its mutant exhibits increased sensitivity to salt stress (Ahmad et al., 2015). *GmbZIP72* interacted with members from the NPR (Nonexpressor of Pathogenesis-Related genes) family, including *NPR1*, *NPR3*, and *NPR5*. Among these, *NPR1* has been demonstrated to improve plant salt stress tolerance (Soyeon et al., 2023). *GmbZIP83* interacted with light signaling-related genes, such as *PIF3* (PHYTOCHROME-INTERACTING FACTOR 3), *COP1* (CONSTITUTIVE PHOTOMORPHOGENIC 1), and *DET1* (DE-ETIOLATED 1). Studies indicate that PIFs act as negative regulators of plant salt tolerance (Ma et al., 2023). The *COP1* mutant exhibits enhanced salt tolerance, a phenotype positively correlated with sucrose accumulation (Kim et al., 2022; Ji et al., 2024). Similarly, the *DET1* mutant also demonstrates increased salt tolerance and shows a synergistic phenotypic effect with *COP1* (Fernando and Schroeder, 2016). The five genes annotated as *DPBF3* (*GmbZIP17*, *GmbZIP42*, *GmbZIP79*, *GmbZIP84*, and *GmbZIP90*) primarily interacted with *SnRK2* (SNF1-RELATED KINASE 2) genes, including *SRK2A*, *SRK2B*, *SRK2D*, *SRK2E*, *SRK2F*, and *SRK2I*. Members of this family have been confirmed to participate in salt stress responses across diverse plant species (Kawa et al., 2020; Liu et al., 2024a, Liu et al., 2024b).

**Figure 11:**
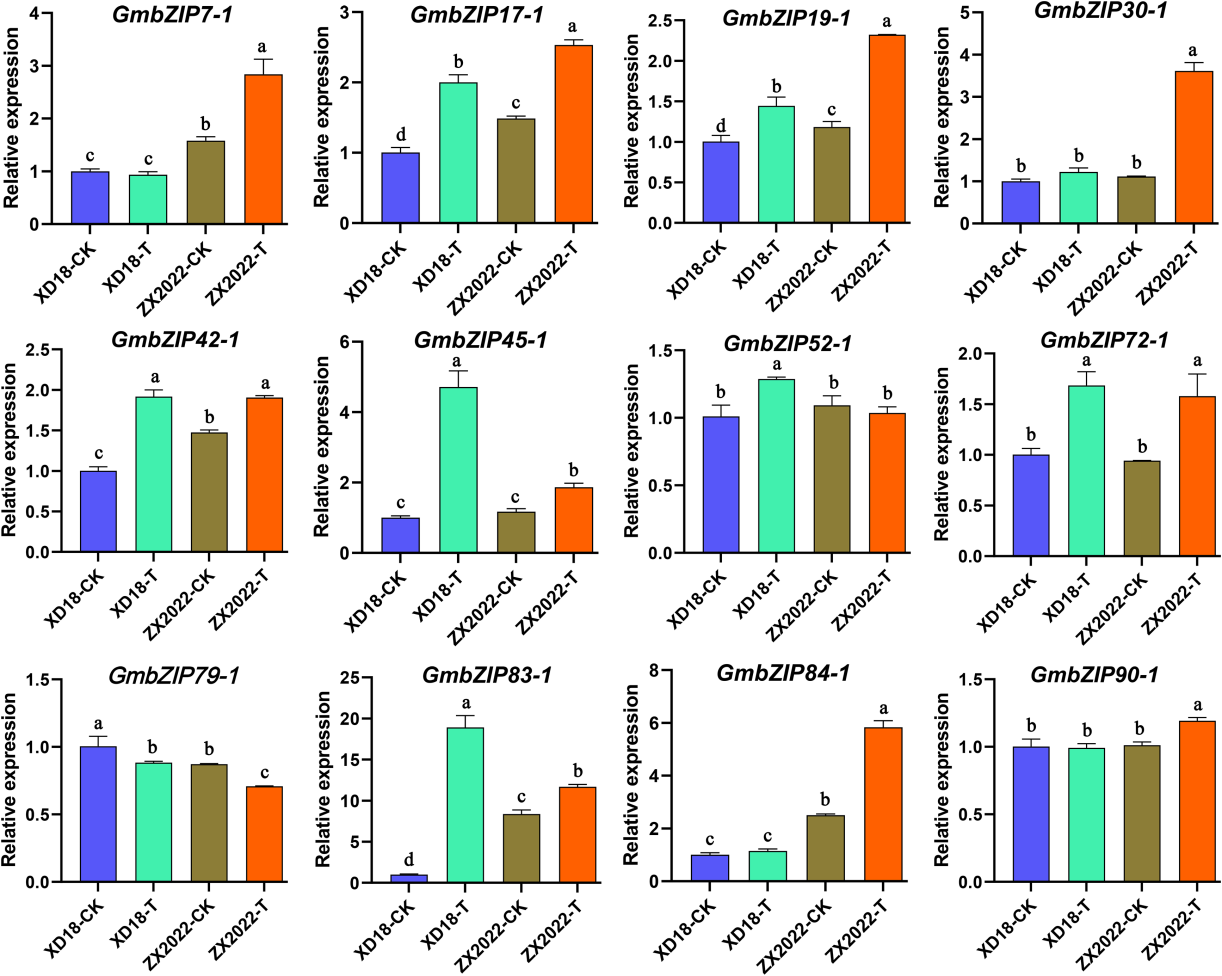
Expression patterns of *GmbZIP* genes under salt stress via qRT-PCR analysis. In this experiment, a salt stress treatment group (T) and a control group (CK) were established. Statistical differences among the four experimental groups (XD18-CK, XD18-T, ZX2022-CK, ZX2022-T) were denoted by lowercase letters (a-d), where different letters indicate significant differences (P < 0.05) and the same letter represents no significant difference. The statistical significance analysis was performed using one-way ANOVA in SPSS software.

To elucidate the transcriptional response mechanisms of candidate *GmbZIP* genes involved in the salt stress response, this study analyzed the relative expression levels of 12 candidate *GmbZIP* genes in the salt-tolerant cultivar XD18 and the salt-sensitive material ZX2022. The results showed that after salt stress treatment, the expression of 11 *GmbZIP* genes was upregulated to varying degrees, with the exception of *GmbZIP79−1*, and the 12 candidate genes exhibited distinct expression patterns in the two genotypes. In XD18, most *GmbZIP* genes exhibited significant transcriptional regulation in response to salt stress. Compared with the control condition (XD18−CK), *GmbZIP17−1*, *GmbZIP19−1*, *GmbZIP42−1*, *GmbZIP45−1*, *GmbZIP52−1*, *GmbZIP72−1*, *GmbZIP79−1*, and GmbZIP83−1 all demonstrated significant up− or downregulation under salt stress treatment (XD18−T). For example, the expression levels of *GmbZIP83−1* and *GmbZIP45−1* in XD18−T were significantly higher than those in XD18−CK. In contrast, the expression responses of these genes to salt stress were weaker in the salt−sensitive material ZX2022. For instance, although the expression of *GmbZIP83–1* was significantly upregulated under salt stress in ZX2022, the magnitude of its regulation was markedly lower than that in XD18. Furthermore, comparative analysis between the two genotypes revealed differences in the expression levels of several *GmbZIP* genes between XD18 and ZX2022 under both control (CK) and treatment (T) conditions. For example, the expression levels of *GmbZIP17−1* and *GmbZIP19−1* in XD18 (under both CK and T) were lower than those in ZX2022, while *GmbZIP79−1* showed the opposite trend. In summary, these findings indicate that all 12 candidate *GmbZIP* genes are involved in the salt stress response, and their differential transcriptional regulation in XD18 and ZX2022 may be an important reason for the difference in salt tolerance between the two genotypes (**Figure 11**).

## 4 Discussion

The bZIP gene family is one of the largest and most functionally complex gene families in plants. It is widely involved in various growth and development processes, such as seed development, as well as in responses to abiotic stresses. In-depth exploration of its biological functions holds significant potential for improving soybean yield and stress tolerance. To date, the bZIP family has been identified and functionally characterized in multiple plant species. The number of bZIP members varies among different species, this variation is not determined by genome size or taxonomic group, but rather by genomic ploidy level and duplication patterns (Rahman et al., 2023). Alternative splicing, as a crucial post-transcriptional regulatory mechanism, enables the generation of multiple transcript isoforms from a single pre-mRNA, thereby significantly expanding the coding potential of the genome (Kornblihtt et al., 2013). Based on whole-genome data of soybean, a total of 171 *GmbZIP* genes were identified, corresponding to 398 proteins, among these, 385 proteins contain the bZIP domain, while the remaining 13 may have lost this domain due to alternative splicing (**Supplementary Table 2**). This phenomenon reflects a strategy of plants to enhance gene function and environmental adaptability through transcript diversity (Xu et al., 2020). Furthermore, by integrating transcriptomic data from soybean under abiotic stress conditions, 92 *GmbZIP* genes potentially associated with stress responses were screened. This represents the first genome-wide systematic identification and evolutionary analysis of *GmbZIP* genes related to abiotic stress in soybean. By constructing a phylogenetic tree of these genes with the *Arabidopsis* bZIP family and classifying them into 13 subfamilies based on the *Arabidopsis* classification system. The results showed that subfamilies B, F, and M do not contain *GmbZIP* genes. Existing literature indicates that genes in these three subfamilies are primarily involved in plant adaptation to zinc deficiency and the regulation of endoplasmic reticulum stress responses (Dröge-Laser et al., 2018).

To systematically elucidate the expression patterns and functions of these 92 *GmbZIP* genes, this study further conducted analyses of expression trends, expression profiles, promoter cis-acting elements, and KEGG pathway enrichment. KEGG annotation results revealed that 48 *GmbZIP* genes were significantly enriched in five pathways, with the plant hormone signal transduction pathway containing the highest number of genes (accounting for 66.7%) and showing the greatest significance. This finding is highly consistent with the promoter cis-acting element analysis: plant hormone-responsive elements were detected in the promoter regions of 91 *GmbZIP* genes (accounting for 96.8%). As endogenous signaling molecules, plant hormones play pivotal roles in abiotic stress responses, among which the SA, ethylene (ETH), ABA, and MeJA are regulated by bZIP transcription factors. Previous studies have shown that overexpression of potato *StbZIP-65* enhances salt tolerance in *Arabidopsis*, and its promoter activity is induced by MeJA (Zhao et al., 2020). Maize *ZmbZIP4* promotes ABA biosynthesis, thereby improving plant adaptation to drought and salt stress (Ma et al., 2018). Moreover, chromatin immunoprecipitation assay analysis confirmed that *GmbZIP60* directly binds to the promoters of abiotic stress-related genes induced by ABA,ETH, JA, and SA, thereby regulating soybean responses to drought and salt stress (Chai et al., 2025).

Phylogenetic analysis found an interesting phenomenon: within subfamily I, a distinct branch exclusively clustered nine soybean *GmbZIP* genes (*GmbZIP21*, *GmbZIP26*, *GmbZIP32*, *GmbZIP35*, *GmbZIP41*, *GmbZIP45*, *GmbZIP53*, *GmbZIP58*, and *GmbZIP78*), with no *Arabidopsis* bZIP homologs present. Further sequence alignment and structural annotation indicated that these genes encode both a Homeodomain and a bZIP domain (**Supplementary Figure 2**), classifying them as mixed-domain transcription factors. Such transcription factors, as “advanced intelligent regulatory units” formed during plant evolution, overcome the functional limitations of single-domain factors. They achieve a “1 + 1 > 2” synergistic regulatory effect through domain fusion, serving as a key molecular basis for plants to adapt to complex and variable environments and coordinate multiple biological processes. Consequently, they represent important bridges connecting genomic information with complex phenotypic traits. Additionally, the study found that *GmbZIP3* and *GmbZIP89* were annotated as GBF1. In *Arabidopsis*, the *GBF1* gene is classified under subfamily G, whereas in soybean, these two genes are clustered within subfamily S. This classification discrepancy may arise from sequence variations during species evolution or from their high homology with genes in subfamily S. To further clarify their evolutionary affiliation, we performed a clustering analysis of *GmbZIP3*, *GmbZIP89* together with the *bZIP* genes from subfamilies S and G of *Arabidopsis*. The results showed that *GmbZIP3* and *GmbZIP89* correctly clustered within the same branch as *Arabidopsis* subfamily G genes (**Supplementary Figure 3**), thereby supporting that their functional and evolutionary classification still belongs to subfamily G.

Based on bioinformatics analyses including phylogenetic tree clustering, collinearity map construction, and BLAST alignment, this study identified 12 candidate *GmbZIP* genes potentially involved in salt stress responses in soybean. Existing research indicates that these genes not only function in salt stress regulation but also participate broadly in responses to other abiotic stresses. For example, overexpression of *GmbZIP7* (*Glyma.02G131700*) enhances soybean tolerance to high salinity, low temperature, and drought (Gao et al., 2011; Zhang et al., 2025). Overexpression of *GmbZIP19* (*Glyma.04G039300*) reduces endogenous ABA levels in plants, leading to decreased waterlogging tolerance (Lin et al., 2024), while this gene can also enhance drought resistance by regulating ABA signaling and the antioxidant defense system (Li et al., 2024a). Overexpression of *GmbZIP83* (*Glyma.18G117100*) significantly suppresses plant height under shading stress and physically interacts with the promoter regions of *GmGA2ox7a* and *GmGA2ox7b* (Lyu et al., 2021). Furthermore, among the 92 identified abiotic stress-related *GmbZIP* genes, multiple members have been demonstrated to play key roles under various stresses. For instance, *GmbZIP5* (*Glyma.02G012700*) and *GmbZIP85* (*Glyma.19G122800*) are differentially expressed under waterlogging stress, and *GmbZIP31* (*Glyma.06G048500*) is induced under shading stress. Studies have confirmed that overexpression of these three genes enhances soybean tolerance to both salt and drought stress (Li et al., 2017; Yang et al., 2020; Chai et al., 2025). *GmbZIP37* (*Glyma.08G115300*) shows differential expression under both waterlogging and salt stress, and its overexpression improves plant salt tolerance (Xu et al., 2016). *GmbZIP8* (*Glyma.02G161100*) is upregulated under salt stress and has been shown to negatively regulate soybean salt and drought tolerance (Zhang et al., 2020). These results collectively highlight the important functions of *GmbZIP* genes in abiotic stress responses and underscore their potential value in breeding stress-resistant crops. However, a comprehensive understanding of the molecular regulatory networks of these 92 candidate *GmbZIP* genes under stress conditions requires further in-depth investigation.

## 5 Conclusion

In summary, this study identified 171 *GmbZIP* genes from the soybean genome and screened 92 stress-responsive members based on transcriptomic data from waterlogging, salt, and shading stress conditions. Phylogenetic analysis classified these genes into 10 subfamilies. Analyses of gene structure, conserved domains, and conserved motifs revealed that each subfamily has distinct compositional characteristics. Further genome-wide alignment and collinearity analysis identified 12 *GmbZIP* genes potentially involved in salt stress regulation, which was also supported by PPI network predictions and qRT-PCR expression analyses. These findings provide an important foundation for future in-depth exploration of the functions and mechanisms of *GmbZIP* genes in soybean abiotic stress responses.

## Data Availability

The datasets presented in this study can be found in online repositories. The names of the repository/repositories and accession number(s) can be found in the article/**Supplementary Material**.
