# Supplementary material for: Global analysis of soybean bZIP gene family under stress and identification of salt-stress-responsive candidate genes
Source: Front Plant Sci. 2026 Mar 20;17:1806221. doi: 10.3389/fpls.2026.1806221 (PMC13047201; doi:10.3389/fpls.2026.1806221)
Supplement: Supplementary file 1 [file Table1.docx]

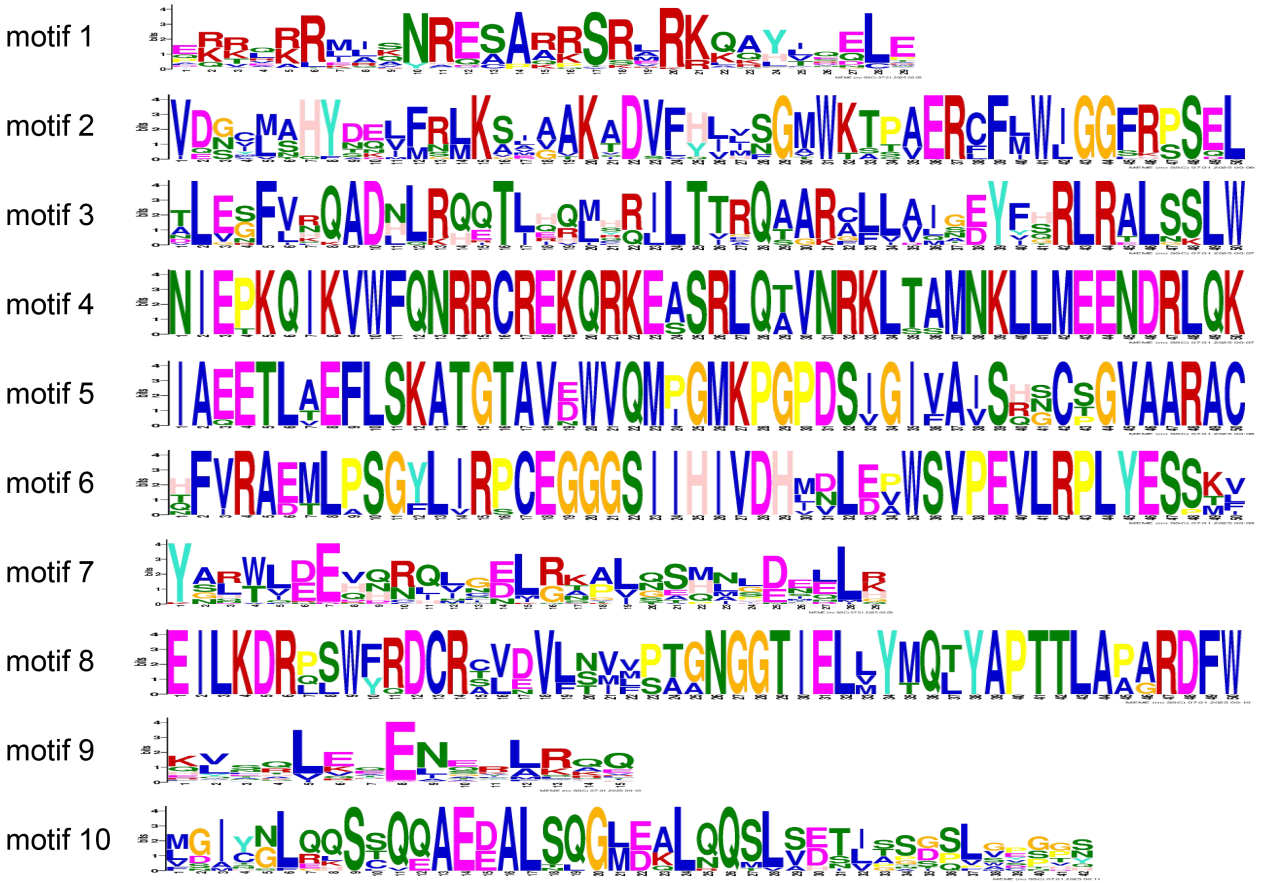


Supplementary Figure S1 Information of 10 conservative motifs of 92 GmbZIP proteins.


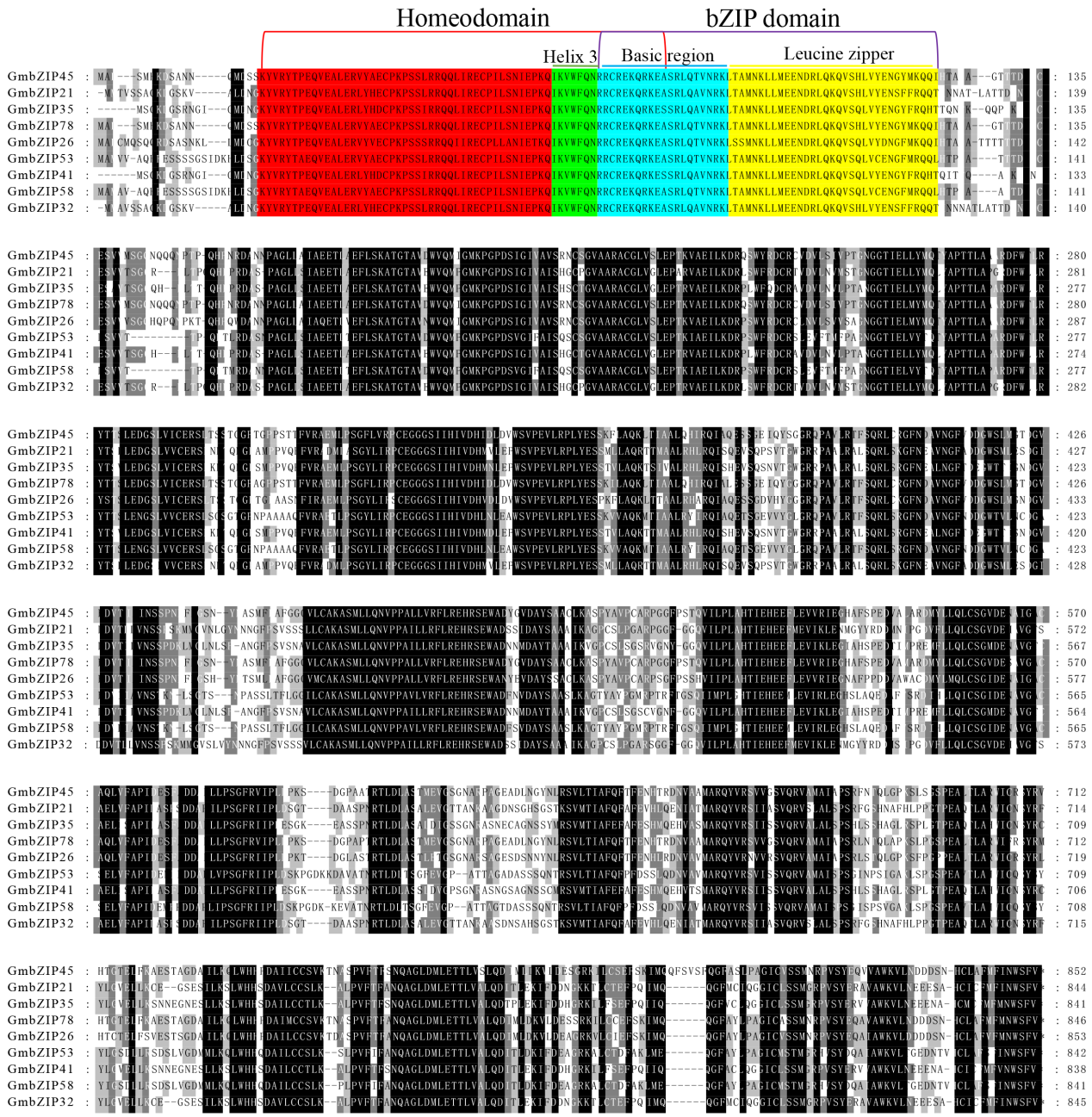


Supplementary Figure S2 Protein sequence alignment of *GmbZIP* gene containing both Homeodomain and bZIP domains.


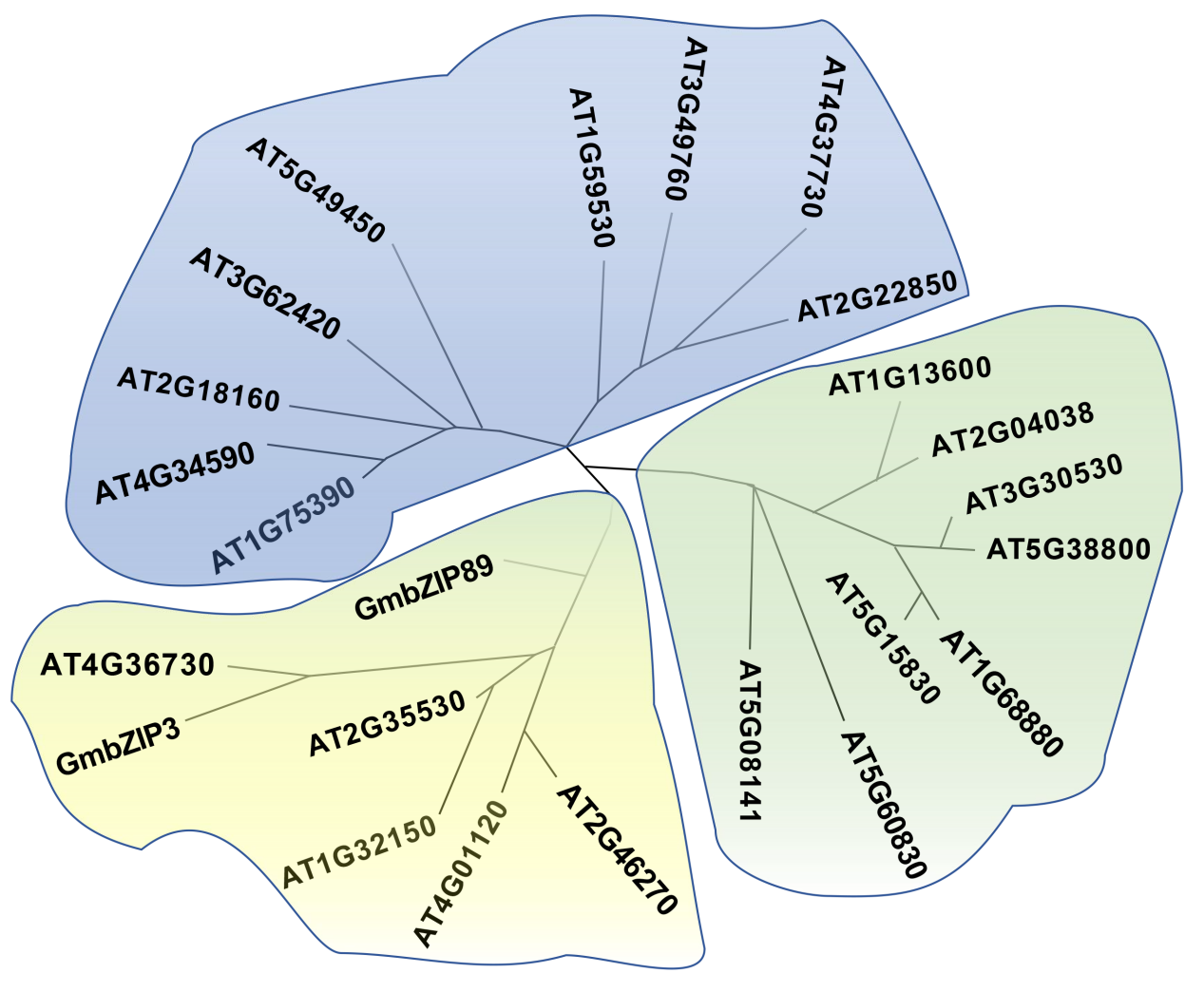


Supplementary Figure S3 Phylogenetic analysis of soybean *GmbZIP3* and *GmbZIP89* alongside Arabidopsis *AtbZIP* genes from the S and G subgroups.
